# Enterotoxigenic *Escherichia coli* heat-labile enterotoxin induces cell death and disrupts effector functions in porcine monocytes

**DOI:** 10.1186/s13567-025-01540-w

**Published:** 2025-07-06

**Authors:** Jinglin Ma, Hans Van der Weken, Leen Hermans, Matthias Dierick, Eric Cox, Bert Devriendt

**Affiliations:** Laboratory Immunology, Department of Translational Physiology, Infectiology and Public Health, Faculty of Veterinary Medicine, Ghent University, Merelbeke, Belgium

**Keywords:** Heat labile enterotoxin, heat stable enterotoxin, monocytes, immune evasion, pig

## Abstract

Enterotoxigenic *Escherichia coli* (ETEC) is a common cause of diarrhea in humans and animals, including pigs. Enterotoxins are important virulence factors for ETEC. Although much is known about the mechanism of enterotoxin-induced diarrhoea, less is known about its effects on innate immune cells such as monocytes. Monocytes can differentiate into macrophages and dendritic cells and play a pivotal role in bridging the innate and adaptive immune systems. Understanding the interaction between ETEC enterotoxins and monocytes can help in the development of more effective preventive and therapeutic strategies to combat this disease. In this study, we aimed to investigate the effects of heat labile enterotoxin (LT) and heat stable enterotoxin a (STa) produced by ETEC on porcine monocytes. Our results showed that STa did not affect the viability or effector functions of monocytes. LT, on the other hand, decreased the viability of monocytes. While LT did not alter the production of reactive oxygen species (ROS) by monocytes, it significantly reduced the production of ROS induced by phorbol 12-myristate 13-acetate (PMA). In addition, LT decreased the phagocytosis of *E. coli* by monocytes and enhanced the survival of intracellular ETEC. Furthermore, LT triggered the production of the cytokines IL-1β, IL-6 and TNF-α as well as the chemokines CCL-3 and CXCL-8. Together, our results show that, in contrast to STa, LT can cause monocyte death and disrupt monocyte immune effector functions, potentially acting as an immune evasion strategy to establish infection.

## Introduction

Enterotoxigenic *Escherichia coli* (ETEC) is a common cause of diarrhea in children and travelers [1]. ETEC infections also cause morbidity and mortality in farm animals, including piglets, leading to significant economic losses to pig farmers. In ETEC-induced diarrhea, the heat labile enterotoxin (LT) and heat stable enterotoxin (ST) secreted by ETEC play crucial roles. Upon secretion, LT enters host gut epithelial cells through binding with ganglioside M1 (GM1). Once internalized, LT activates adenylate cyclase, which results in an increase in the level of intracellular cyclic adenosine monophosphate (cAMP). The latter in turn activates protein kinase A (PKA), which modulates the activation of membrane ion channels, ultimately leading to the secretion of electrolytes and water into the intestinal lumen [2, 3]. To date, two distinct types of STs, namely, STa and STb, have been identified. The initial phase of STa-induced diarrhoea involves the activation of guanylate cyclase C (GC-C) on gut epithelial cells. This leads to increased cyclic guanosine monophosphate (cGMP) levels and subsequent activation of cGMP-dependent protein kinase II (cGMPKII). These molecular cascades induce the secretion of chloride and bicarbonate ions while inhibiting Na^+^ uptake, thereby causing diarrhoea [3].

ETEC strains produce STs that not only induce secretory diarrhea but also modulate the expression of proinflammatory cytokines, chemokines, and other immune-related genes [4]. Moreover, their impact on the innate immune function of enterocytes has been explored recently. For example, STa induces rapid and transient expression of the interleukins IL-33 and IL-1Ra in human gut epithelial cells [5]. Additionally, compared with wild-type mice, mice deficient in the IL-33 receptor are less susceptible to STa [6]. However, the impact of STs on the immune function of intestinal immune cells remains largely unexplored. In contrast to STs, LTs have been reported to activate human and murine immune cells and to enhance cellular and humoral immune responses to antigens [7]. In-depth investigations have elucidated the underlying mechanisms involved. Some studies in mice have indicated that the enhancement of T-cell proliferation is linked to the functional activation of dendritic cells induced by LT [8, 9]. However, previous results suggest that the mechanism is complex and that various immune cells are involved in this immunomodulation process. For example, in a mouse model, LT administration increased the production of IL-1β by dendritic cells [10]. On the other hand, another study indicated that LT-IIa and LT-IIb could suppress the production of IL-1β induced by LPS in the human monocytic cell line THP-1 [11]. Furthermore, previous studies have indicated that LT can increase antigen uptake by murine dendritic cells [12, 13], whereas a recent study demonstrated that LT inhibits the phagocytosis of ETEC by murine macrophages [14]. Although the effect of LT on certain immune cell populations is well established, its effect on monocytes, precursors to macrophages and dendritic cells, remains poorly understood, particularly in pigs.

Monocytes play pivotal roles as bridges between the innate and adaptive immune systems. Like various pattern recognition receptors, they can detect both pathogen-associated molecular patterns (PAMPs) and damage-associated molecular patterns (DAMPs) [15]. Upon recognizing these signals, monocytes become activated and undergo chemotaxis, migrating towards the infection site. Once they are present, they exhibit a certain degree of plasticity by differentiating into either macrophages or dendritic cells, which are influenced by local cues and signals. Moreover, even under steady-state conditions, there is a constant influx of blood monocytes into the gut, where they continuously replenish intestinal macrophages and dendritic cells [16]. This versatility allows monocytes to contribute to gut homeostasis [17]. Some of these monocytes, however, retain their characteristics and reside in the intestinal lamina propria [18]. In addition, monocytes are present within the small intestinal lumen in mice. When ETEC enters the small intestine or when the epithelial barrier is disrupted by ETEC [19], these resident monocytes are exposed to ETEC and its secreted enterotoxins. In response, monocytes engage their effector functions, including phagocytosis, reactive oxygen species (ROS) production, and the release of cytokines and chemokines, to eliminate pathogens [20]. Moreover, monocytes can serve as antigen-presenting cells, possessing the ability to capture, process, and deliver antigens to T cells, thereby facilitating the coordination of the host immune response [16]. Here, we investigated the effects of LT and STa on the function of porcine monocytes. These findings contribute to a deeper understanding of immunoregulation by LT and STa.

## Materials and methods

###  Enterotoxins

LT was purified from the supernatant of the ETEC strain IMM07 as previously described [21]. The endotoxin content of LT was measured using the Pierce™ Chromogenic Endotoxin Quant Kit (Thermo Fisher Scientific, Waltham, USA) and was found to be 9.88 ng of LPS per 100 μg of LT.

###  Isolation and culture of monocytes

Monocytes were isolated as described previously [22]. In brief, peripheral blood was collected from 10- to 24-week-old pigs via the jugular vein. The PBMCs were subsequently isolated by density gradient centrifugation on lymphoprep (Axis-Shield, Dundee, UK). Isolated PBMCs were incubated with mouse anti-porcine CD14 antibodies (clone MIL-2, in-house production) for 40 min. After washing, the cells were incubated with secondary goat-anti-mouse IgG Microbeads (Miltenyi, Bergisch Gladbach, Germany) for another 20 min. Subsequently, the cells were diluted in 5 mL of PBS-EDTA + 1% FCS and applied onto an LS column (Miltenyi) to obtain CD14^+^ cells. This cell population had a purity exceeding 95% and a cell viability exceeding 90%, as assessed by flow cytometry (Cytoflex, Beckman Coulter Biosciences, Indianapolis, USA). Purified monocytes were resuspended at a density of 2 × 10^6^ cells/mL in phenol red-free RPMI 1640 medium (Gibco, Waltham, USA) supplemented with 10% foetal calf serum (FCS, Gibco) and 1% penicillin/streptomycin (Gibco). The pigs (females, 10- to 24-week-old) used as blood donors were housed under standard conditions. All animal experiments were approved by the animal care and ethics committee of the Faculty of Veterinary Medicine, Ghent University (EC2017/121; EC2023/22).

###  LT binding assay

The binding of LT to the membrane of monocytes was assessed using flow cytometry. Monocytes were seeded at 2 × 10^5^ cells per well in 96-well plates and incubated at 37 °C with 5% CO_2_ for 1 h. Subsequently, the monocytes were incubated with 0, 20, or 500 ng/mL LT for an additional 30 min on ice. After incubation, the monocytes were washed three times with ice-cold PBS and then incubated for 3 h on ice with a rabbit anti-*E. coli* LT polyclonal antibody (1:300, Abcam, Cambridge, UK) in ice-cold PBS containing 1% FCS. Following three washes with ice-cold PBS, the monocytes were stained for 1 h on ice with a PE-labelled goat anti-rabbit IgG polyclonal antibody (1:300, Invitrogen, Waltham, USA) in ice-cold PBS. After three more washes, the monocytes were stained with the viability dye SYTOX™ blue (1 μM, Invitrogen, Carlsbad, CA, USA) in PBS and analysed by flow cytometry (Cytoflex, Beckman Coulter). The data were analysed using CytExpert software (Beckman Coulter). For the GM1 inhibition assay, 0, 25, or 500 ng of LT was preincubated with 1 μg of GM1 (Sigma, Saint Louis, USA) for 2 h at 37 °C before being added to the monocytes.

###  cAMP ELISA

Monocytes were cultured in a 24-well plate at a density of 2 × 10^6^ cells/well. After a 1 h incubation period, monocytes (2 × 10^6^ cells/well) were subsequently pretreated with either LT (0, 20 or 500 ng/mL) for another 1 h at 37 °C. After centrifugation, the cell supernatants were collected, and monocytes were lysed with 0.1 M HCl to stop endogenous phosphodiesterase activity. After centrifugation at 660 × *g* for 10 min at room temperature to remove cellular debris, the cell lysates were collected. The cAMP level in the monocyte supernatants and lysates was assayed using a Direct cAMP ELISA Kit (Enzo Life Sciences, New York, USA) following the manufacturer’s guidelines.

###  Cell viability assay

To assess the viability of monocytes, we used propidium iodide (PI; Sigma) to stain the cells. Monocytes (2 × 10^5^ cells/well) were seeded into 96-well plates and then incubated at 37 °C in 5% CO_2_ for 1 h to allow the monocytes to settle. Subsequently, the monocytes were treated with 0, 4, 20, 100 or 500 ng/mL LT or 0, 100 or 500 ng/mL STa for 4 h or 24 h. Next, the monocytes were harvested and transferred to a 96-well V-bottom plate. They were then resuspended in a 1 μg/mL PI solution. The cell viability was subsequently analysed using a flow cytometer from Beckman Coulter, and the resulting data were analysed using CytExpert software (Beckman Coulter). Doublets were excluded on the basis of FSC-H/FSC-A and SSC-H/SSC-A. A minimum of 10 000 events were counted for each analysis.

###  Phagocytosis assay

Monocytes (2 × 10^5^ cells/well) were pretreated with either LT (0, 4 20, 100 or 500 ng/mL) or STa (0, 100 or 500 ng/mL) for 2 or 8 h at 37 °C. Next, 6 × 10^6^ pHrodo™ red-labelled *Escherichia coli* particles (Invitrogen) were added to the cells, which were then incubated at 37 °C for an additional 2 h. Subsequently, the monocytes were carefully collected in a 96-well V-bottom plate and washed three times with cold PBS to remove free-floating *E. coli* particles. Then, the monocytes were resuspended in 100 μL of PBS containing Sytox™ Blue (1 μM, Invitrogen) and incubated for 10 min at 4 °C. Finally, the monocytes were analysed using flow cytometry (Beckman Coulter), and the resulting data were analysed using CytExpert software (Beckman Coulter).

To measure the effects of LT on the survival of ETECs phagocytosed by porcine monocytes, isolated monocytes were added to a 96-well plate at a density of 3 × 10^5^ cells/well. After a rest period at 37 °C for 2 h, monocytes were pretreated with LT (0, 100 or 500 ng/mL) for 24 h at 37 °C. Next, 9 × 10^5^ of ETEC was added to the cells, which were then incubated at 37 °C for an additional 2 h to allow phagocytosis. In this assay, three ETEC strains were utilized: GIS26 (O149:K91, F4ac, LT^+^STa^+^STb^+^) [23], the LT deletion mutant GIS26 (GIS26ΔLT, O149:K91, F4ac, LT^−^STa^−^STb^+^) [23] and 2134P (O157, F18ac, LT^−^STa^+^STb^+^) [24]. The monocytes were subsequently collected, transferred to a 96-well V-bottom plate and washed three times with cold PBS to remove free-floating ETEC. Then, the monocytes were resuspended in 100 μL of RPMI 1640 medium containing 100 µg/mL gentamicin and incubated for 1 h at 37 °C to kill the extracellular bacteria. After being washed 3 times with cold PBS to remove gentamicin, the monocytes were lysed with 200 μL of distilled water at room temperature. Finally, 10 μL of the lysate containing intracellular ETEC was plated on brain heart infusion (BHI, Thermo Fisher Scientific) agar to quantify the number of surviving ETEC.

###  Analysis of reactive oxygen species (ROS) production

We conducted a luminol chemiluminescence assay to assess the production of reactive oxygen species (ROS) by monocytes under two experimental conditions. To measure the level of ROS production induced by LT, STa and ETEC, monocytes (2 × 10^5^ cells/well) were plated in a 96-well white microplate. After a 1 h incubation period, we replaced the culture medium with 175 μL of a luminol solution at 100 μg/mL. Subsequently, 25 μL of either LT (0, 32, 160, 800 or 4000 ng/mL), STa (0, 800 or 4000 ng/mL) or ETEC (GIS26ΔLT; 1.6 × 10^8^, 1.6 × 10^7^ or 1.6 × 10^6^ CFU/mL) was added, following a 5 min background measurement. Chemiluminescence was continuously monitored at 5 min intervals for a total of 2 h at 37 °C via Luminoskan Microplate Readers (MTX Lab system, Vienna, USA). As a positive control, monocytes were stimulated with 50 μg/mL phorbol myristate acetate (PMA, Sigma).

To investigate the effects of LT and STa on PMA-, MacroGard® β-glucan (Biotec Pharmacon ASA, Tromsø, Norway)- and ETEC-induced ROS production, monocytes were first pretreated with LT or STa for either 2 or 8 h. Subsequently, the culture medium was replaced with 175 μL of a 100 μg/mL luminol solution to measure the background for 5 min, followed by the addition of 25 μL of the PMA (400 μg/mL), β-glucan (4 mg/mL) or GIS26ΔLT (1.6 × 10^8^ CFU/mL) ETEC solution. Chemiluminescence was also recorded at 5 min intervals over a 2 h period at 37 °C.

###  RT‒qPCR

Porcine monocytes were stimulated with LT (0, 20 or 500 ng/mL) or STa (0 or 500 ng/mL) for either 2 or 8 h. Following treatment, monocytes were harvested, and total RNA was extracted by the Qiagen Shredder and RNeasy Mini Kit from Qiagen, which adheres to the manufacturer's guidelines. The RNA quantity and purity were determined via microvolume UV‒Vis spectrophotometry (DeNovix, Wilmington, USA), whereas the RNA integrity was assessed by running samples on denaturing agarose gels stained with ethidium bromide (EtBr, Sigma). To remove potential genomic DNA contamination, RNA (500 ng) was treated with RQ1 RNase-Free DNase (Promega, Madison, USA). The treated RNA was subsequently reverse transcribed into cDNA via the SuperScript III Reverse Transcriptase Kit from Invitrogen, which was supplemented with a ribonuclease inhibitor (RNase OUT; Invitrogen) according to the manufacturer's instructions. The resulting cDNA served as a template for quantitative polymerase chain reaction (qPCR) assays. The primers (Table 1) were designed with Primer-BLAST (NIH, USA) or taken from the literature and synthesized by Integrated DNA Technologies (IDT, Coralville, IA, USA). The amplification efficiencies ranged between 99 and 102%. Quantitative PCR was carried out using 25 ng of cDNA template, primers at 250 nM, and an annealing temperature of 60 ℃ on a StepOnePlus real-time PCR system (Applied Biosystems, Waltham, USA) and SYBR Green master mix (Applied Biosystems) in a total volume of 20 μL, following the manufacturer’s protocol. Data analysis was performed using the double delta threshold cycle method [25], with normalization to the expression levels of reference genes (β-actin and GAPDH) and control conditions. The selection of reference genes was based on geNorm analysis using qBase + software.

**Table 1:** **Sequences of the primers used in the qPCR assay**.

Target	Accession number	Primer Sequence	References
β-actin	AY550069	**Fw:** TCATCACCATCGGCAACG	[58]
		**Rv:** TTCCTGATGTCCACGTCGC	
GAPDH	AF017079	**Fw:** GGGCATGAACCATGAGAAGT	[59]
		**Rv:** AAGCAGGGATGATGTTCTGG	
IL1β	NM_214055.1	**Fw:** AGCCCAATTCAGGGACCCTAC	–
		**Rv:** TGCCTGATGCTCTTGTTCCA	
IL6	NM_214399.1	**Fw:** CCTGAGATTGATGCCGTCCA	–
		**Rv:** TCTTCAAGCCGTGTAGCCAT	
TNFα	NM_214022	**Fw:** ACTGCACTTCGAGGTTATCGG	[22]
		**Rv:** GGCGACGGGCTTATCTGA	
CXCL8	NM_213867.1	**Fw:** GACCCCAAGGAAAAGTGGGT	–
		**Rv:** TGACCAGCACAGGAATGAGG	
CCL2	NM_214214.1	**Fw:** CCAGGACTCCATAAGCCACC	–
		**Rv:** CAATGTGCCCAAGTCTCCGT	
CCL3L1	NM_001009579.1	**Fw:** CCTCGCAAATTCGTAGCCGA	–
		**Rv:** TCAGCTCCAGGTCAGAGATGT	
CCL5	NM_001129946.1	**Fw:** TGCTTCTTGCTCTTGTCCCA	–
		**Rv:** GTGCCAAGGGTCCAAAGTTC	

Fw, forward primer; Rv, reverse primer

###  Cytokine and chemokine ELISA

Monocytes (1 × 10^6^ cells/well) were seeded in a 24-well microplate. These monocytes were then treated with LT (0, 20 or 500 ng/mL) or STa (0 or 500 ng/mL) for 4 or 24 h. Following the treatment period, the supernatant was carefully collected and centrifuged (400 × *g*, 5 min, 4 °C) to remove residual monocytes. The concentrations of IL1β, IL6, CXCL8, and TNFα in the supernatant were then quantified using commercial ELISA kits from R&D Systems (Minneapolis, USA) following the manufacturer’s instructions. The concentrations of CCL2 and CCL3 in the supernatant were then quantified using commercial ELISA kits from Kingfisher Biotech (Saint Paul, USA) according to the manufacturer’s instructions.

###  Statistical analysis

The experimental data are expressed as the mean ± standard deviation (SD). All the statistical analyses were performed with IBM SPSS Statistics 26 (USA). We assessed the homogeneity of variance among groups using Levene’s test, while the normality of the distribution of the data was assessed via the Shapiro‒Wilk test. For comparisons between two groups, a two-tailed paired Student’s *t* test was used. For comparisons of three to five groups, we used one-way ANOVA with Tukey’s multiple comparison test, or a Friedman test was used if normality tests did not pass. Probability values (*P*) of 0.05 or less were considered significant.

## Results

###  Assessing the binding and cytotoxic effects of LT on porcine monocytes

**Figure 1:**
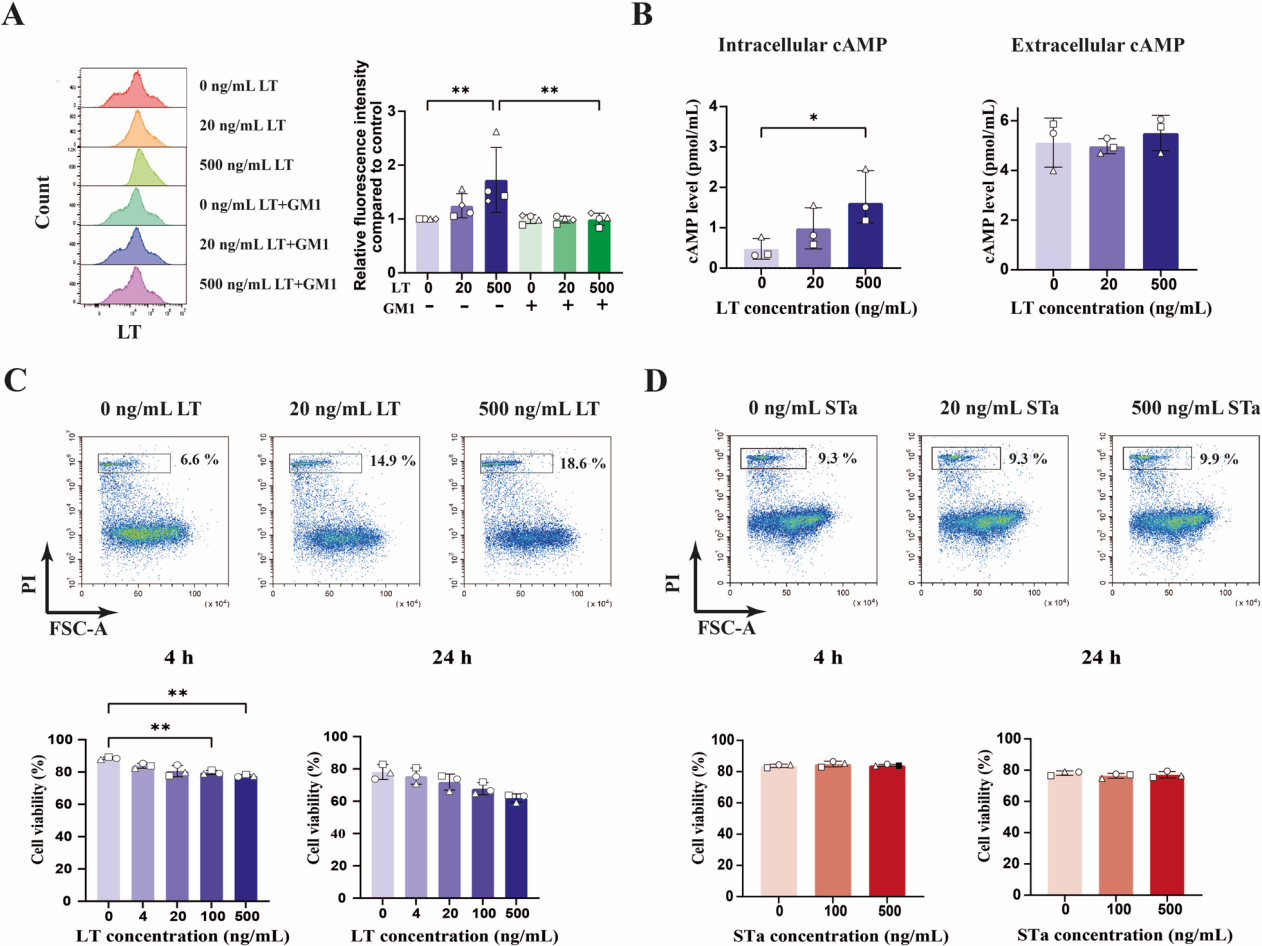
**Assessing the interaction and cytotoxic effects of LT on porcine monocytes.** **A** Monocytes (1 × 10^5^) were incubated with LT or LT pretreated with 1 μg/mL GM1 for 1 h at 4 °C. The binding of LT to the monocyte membrane was analysed by immunostaining and flow cytometry*.* **B** Monocytes (1 × 10^6^) were incubated with 0, 20 or 500 ng/mL LT for 1 h at 37 °C. The cAMP levels in lysed monocytes (left panel) and cell supernatants (right panel) were measured by ELISA. **C**-**D** Representative dot plots of monocyte viability upon treatment with LT or STa for 4 h. Monocytes (2 × 10^5^) were incubated with 0, 4, 20, 100 or 500 ng/mL LT or 0, 100 or 500 ng/mL STa for 4 or 24 h. The treated monocytes were stained with PI and analysed by flow cytometry. *n* = 3 or 4 individual blood donors. The bars represent the mean ± SD. The data from the binding assay were analysed with a Friedman test. The cAMP level and cell viability data were analysed by one-way ANOVA with a post hoc Tukey test to compare the LT or STa treatment groups with the control group. *, *p* < 0.05; **, *p* < 0.01. A paired Student’s *t* test was used to compare two groups with or without GM1. ##, *p* < 0.01.

While type I LT can bind to intestinal epithelial cells via GM1 [26], its binding to porcine immune cells has rarely been investigated. We first explored whether LT could bind to monocytes. Our results demonstrated that LT binds to monocytes and that preincubation of LT with GM1 inhibited this binding (Figure 1A). These findings suggest that GM1 may serve as a receptor for LT on porcine monocytes. In addition to binding to and being internalized by epithelial cells, LT is known to activate adenylate cyclase, leading to cAMP production. Therefore, we measured both intracellular and extracellular cAMP levels following LT treatment. As shown in Figure 1B, LT significantly increased intracellular cAMP production in monocytes but did not affect extracellular cAMP levels. During infection, some pathogens use their virulence factors to induce the death of immune cells, thereby allowing them to evade elimination by the immune system [27, 28]. Thus, we also investigated the cytotoxic effects of LT and STa on monocytes. Figure 1C shows a dose-dependent decrease in cell viability after monocytes were incubated with LT for 4 h. In contrast, STa stimulation for 4 or 24 h did not induce monocyte death (Figure 1D).

###  Effects of LT and STa on phagocytosis and intracellular killing by porcine monocytes

**Figure 2:**
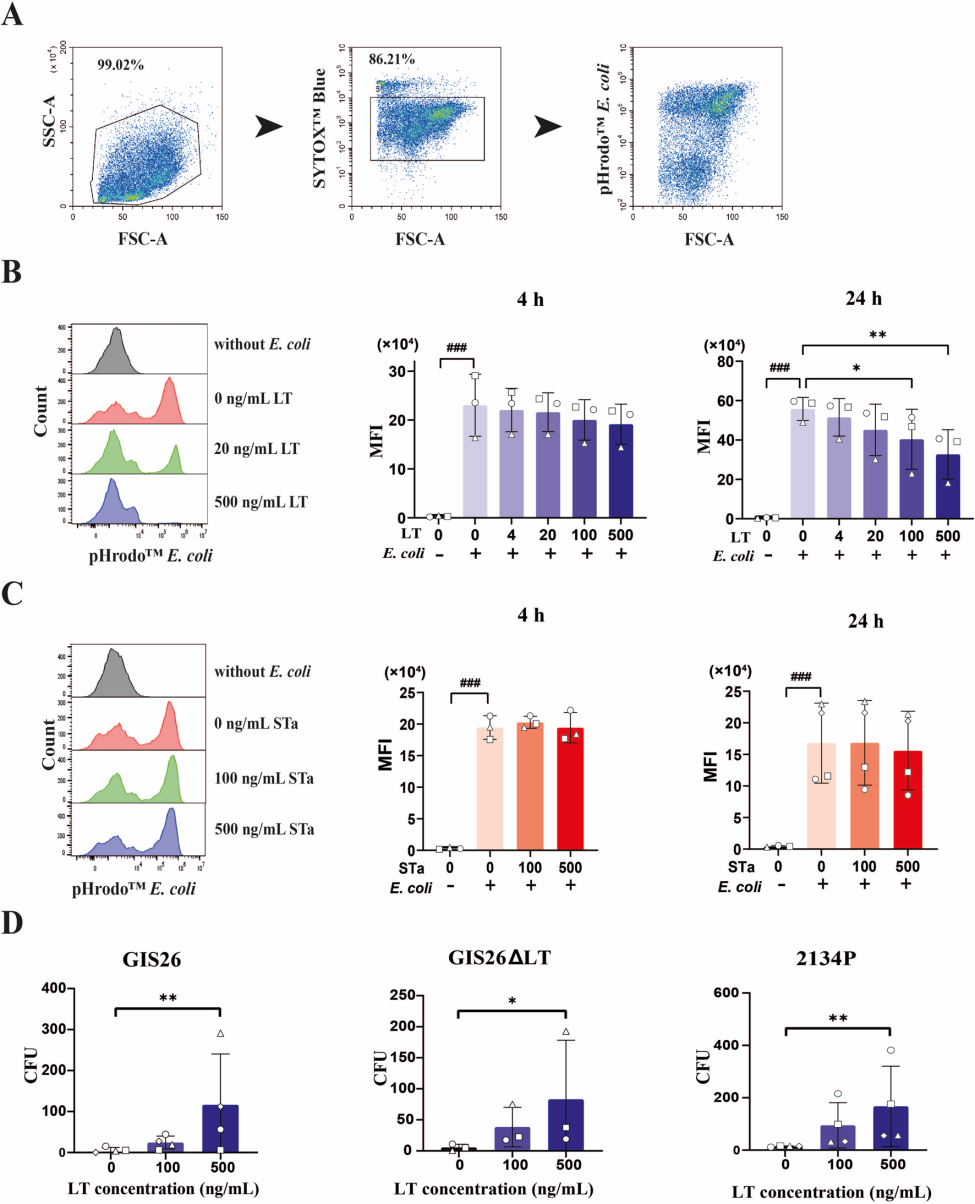
**Effects of LT and STa on phagocytosis and intracellular killing by porcine monocytes.** **A** The gating strategy used to assess the uptake of pHrodo™ by red *E. coli* by flow cytometry. Sytox blue staining was used to exclude dead cells from the analysis. **B**-**C** Representative histograms showing the phagocytosis of pHrodo™ red *E. coli* by monocytes upon incubation with LT (B) or STa (C) for 24 h at the indicated concentrations. Phagocytosis of pHrodo™ red *E. coli* by monocytes (2 × 10^5^) after treatment with LT or STa for 4 or 24 h at the indicated concentrations. MFI: mean fluorescence intensity. **D** Monocytes (3 × 10^5^) were pretreated with 0, 100, or 500 ng/mL LT for 24 h and then incubated with 9 × 10^5^ CFU of ETEC (strains GIS26, GIS26ΔLT, and 2134P) for another 2 h to allow phagocytosis. Extracellular bacteria were then killed with 100 μg/mL gentamicin, and monocyte lysates were plated to quantify viable intracellular bacteria. CFU: colony-forming units. *n* = 3 to 4 individual blood donors. The bars represent the mean ± SD. The data were analysed by one-way ANOVA with a post hoc Tukey test to compare the LT or STa treatment groups to the control group. *, *p* < 0.05; **, *p* < 0.01. A paired Student’s *t* test was used to compare two groups with or without *E. coli*. ###, *p* < 0.001. The data from the ETEC survival assay were analysed with a nonparametric Friedman test *, *p* < 0.05, **, *p* < 0.01.

Phagocytosis is a pivotal function of monocytes in the clearance of pathogens [17]. Recent research has indicated that LT decreases the phagocytic ability of RAW 246.7 cells, a murine macrophage line [14]. Therefore, our study investigated whether LT and STa could influence the phagocytic activity of porcine monocytes. Similarly, we found that LT decreased the phagocytosis of *E. coli* by monocytes at a concentration of 100 or 500 ng/mL after an incubation period of 24 h, as shown in Figures 2A-B. In contrast, STa did not affect the phagocytotic function of monocytes under the tested conditions (Figure 2C). Although phagocytosis is a highly effective mechanism for clearing most pathogens, a large body of evidence suggests that specific virulence factors of some pathogenic bacteria can disrupt this process [29, 30]. To determine whether LT acts as a protective mechanism for ETEC, we examined the survival of intracellular ETEC upon their phagocytosis by primary monocytes. The results revealed that pretreatment of monocytes with 500 ng/mL LT for 24 h significantly reduced their ability to kill all three ETEC strains taken up by monocytes, thereby increasing the survival of intracellular ETEC (Figure 2D).

###  LT but not STa decreases the production of ROS by monocytes

**Figure 3:**
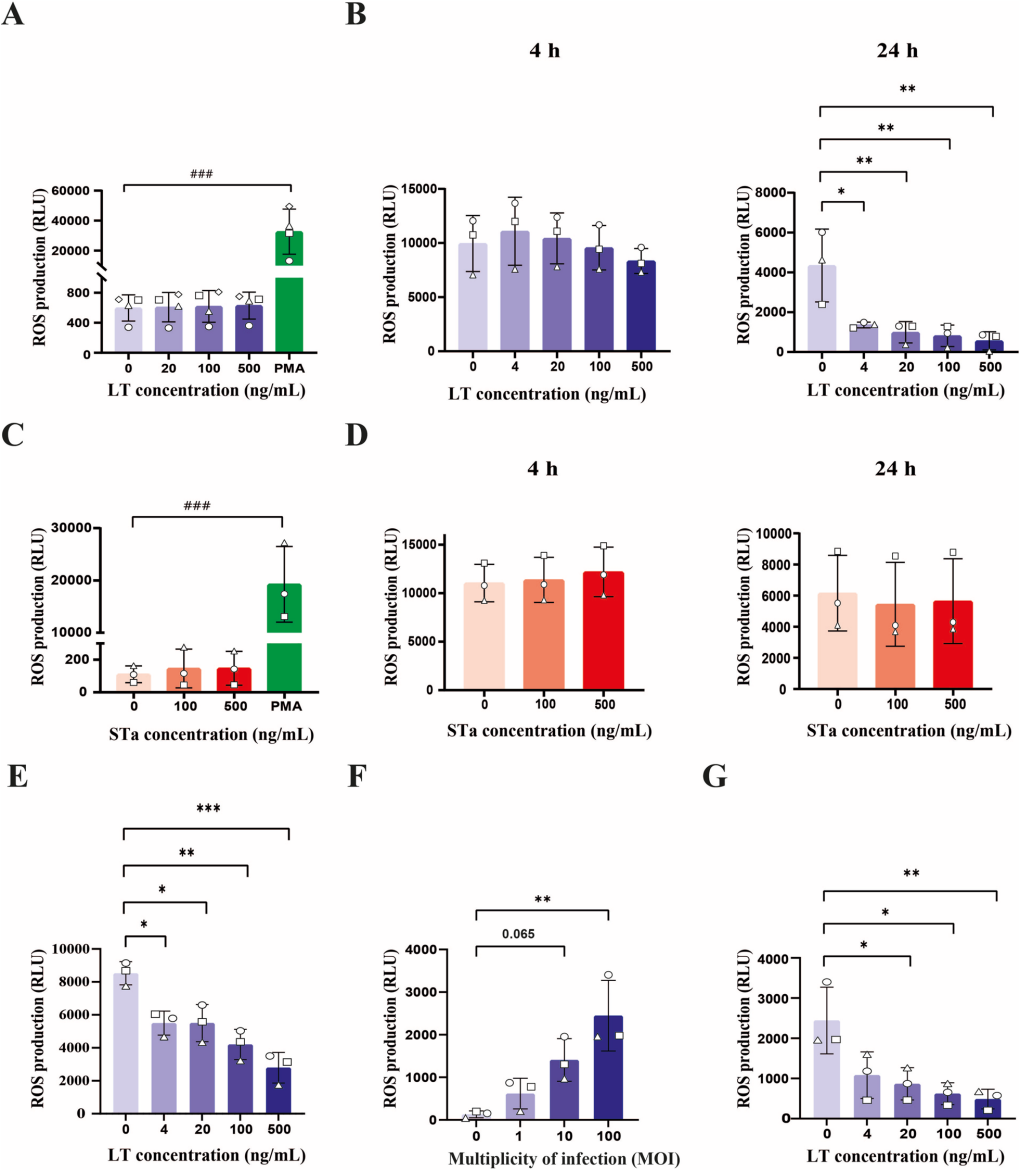
**LT, but not STa, decreased PMA-, β-glucan- and ETEC-induced ROS production by monocytes.** **A** Monocytes (2 × 10^5^) were incubated with 0–500 ng/mL LT or 0–500 ng/mL STa (**C**) or for 2 h at 37 °C. The ROS production of monocytes was analysed by chemiluminescence. RLU: relative light units. **B** Monocytes (2 × 10^5^) were first pretreated with 0–500 ng/mL LT or STa (**D**) for 4 or 24 h at 37 °C and then incubated with 50 μg/mL PMA for another 2 h. **E** Monocytes (2 × 10^5^/well) were first pretreated with 0, 100 or 500 ng/mL LT for 24 h at 37 °C and then incubated with β-glucan (Macrogard; 500 μg/mL) for another 2 h. **F** Monocytes (2 × 10^5^/well) were incubated with 2 × 10^5^, 2 × 10^6^ or 2 × 10^7^ CFUs of GIS26ΔLT for 2 h at 37 °C. The ROS production of monocytes was analysed by chemiluminescence. **G** Monocytes (2 × 10^5^/well) were first pretreated with 0, 100 or 500 ng/mL LT for 24 h at 37 °C and then incubated with 2 × 10^7^ CFUs of GIS26ΔLT for another 2 h. *n* = 3 to 4 individual blood donors. The bars represent the mean ± SD. The data were analysed by one-way ANOVA with a Tukey test to compare the LT or STa treatment groups with the control group. **, *p* < 0.05; **, *p* < 0.01. A paired Student’s *t* test was used to compare two groups with or without PMA. ###, *p* < 0.001.

When exposed to microorganisms, monocytes generate ROS as part of their defense mechanism to eliminate invading pathogens [17]. Therefore, we wondered whether LT or STa could influence ROS production by porcine monocytes. Figures 3A and C show that neither LT nor STa treatment induced ROS production by porcine monocytes. To understand whether LT or STa affect ROS production by monocytes induced by PMA, a known activator of the respiratory burst response in innate immune cells, monocytes were pretreated with the enterotoxins for 4 h or 24 h. However, the ROS production induced by PMA remained unaffected after a 4 h pretreatment with LT or STa; after 24 h, LT reduced PMA-induced ROS production by monocytes, even at the lowest tested concentration (4 ng/mL) (Figures 3B, D). In contrast to LT, STa was unable to alter the ROS production induced by PMA (Figure 3D). To understand whether this effect of LT was specific to PMA, ROS production was induced by β-glucans [31]. As shown in Figure 3E, LT also decreased the ROS production induced by β-glucans in monocytes. We then investigated whether similar effects could be observed in monocytes in response to ETEC. To avoid influences from LT produced by ETEC, we first used an LT deletion mutant ETEC strain (GIS26ΔLT) to assess whether monocytes respond to increased ROS production. As shown in Figure 3F, treatment with this strain significantly increased ROS production by monocytes. However, when monocytes were preincubated with LT, the ROS production induced by ETEC was significantly inhibited (Figure 3G).

###  Evaluation of proinflammatory cytokine and chemokine responses in porcine monocytes upon LT or STa stimulation

**Figure 4:**
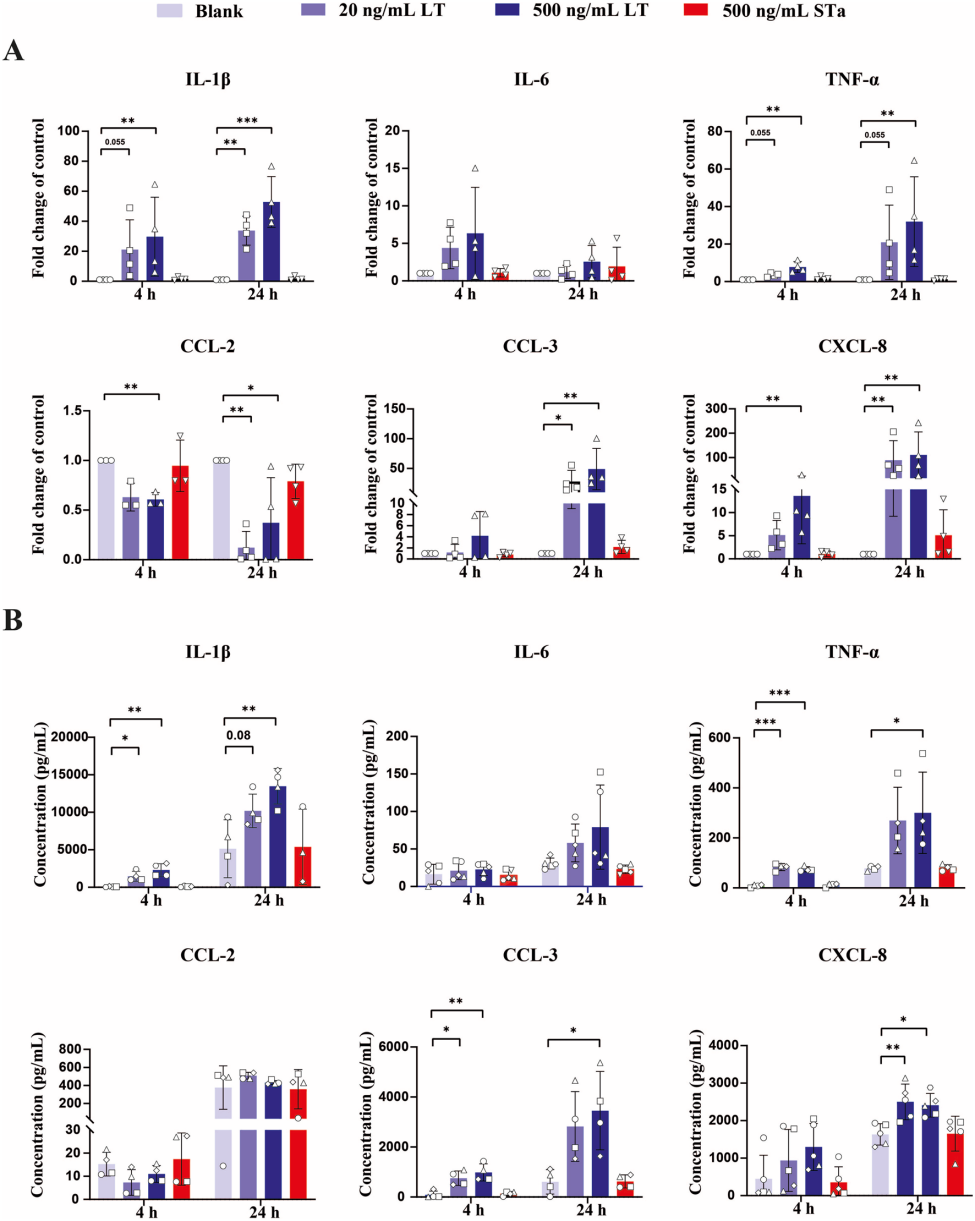
**Evaluation of proinflammatory cytokine and chemokine responses in porcine monocytes upon LT or STa stimulation**. **A** Monocytes (1 × 10^6^) were treated with LT or STa at the indicated concentrations for 4 or 24 h. IL-1β, IL-6, TNF-α, CCL-2, CCL-3 and CXCL-8 transcript levels were evaluated by qPCR. **B** IL-1β, IL-6, TNF-α, CCL-2, CCL-3 and CXCL-8 secretion levels were measured by ELISA in the culture supernatant of monocytes (1 × 10^6^) after treatment with LT or STa at the indicated concentrations for 4 or 24 h. *n* = 3 to 5 individual blood donors. The bars represent the mean ± SD. Data on the mRNA expression of IL1β (4 h), CCL-3 (4 h) and CXCL-8 (24 h) as well as the protein secretion of IL1β (24 h), IL-6 (4 h), TNFα (4 and 24 h), CCL-2 (4 h), CCL-3 (4 and 24 h) and CXCL-8 (4 and 24 h) were analysed by one-way ANOVA with a post hoc Tukey test to compare the LT treatment groups with the control group. Data on the mRNA expression of IL1β (24 h), IL-6 (4 and 24 h), TNFα (4 and 24 h), CCL-2 (4 and 24 h), CCL-3 (24 h) and CXCL-8 (4 h) as well as the protein secretion of IL1β (4 h), IL-6 (24 h) and CCL-2 (24 h) were analysed with a nonparametric Friedman test to compare the LT treatment groups with the control group. A paired Student’s *t* test was used to compare the STa treatment group to the control group. *, *p* < 0.05; **, *p* < 0.01; ***, *p* < 0.001.

In response to perceived danger signals, monocytes can initiate and drive inflammatory responses by releasing a wide range of cytokines and chemokines [17]. In this study, we assessed the expression of key inflammatory mediators, such as IL-1β, IL-6, TNF-α, CCL-2, CCL-3, and CXCL-8, which play important roles in immune cell migration and activation. LT treatment resulted in a dose-dependent increase in the mRNA expression of IL-1β, IL-6, TNF-α, CCL-3 and CXCL-8 after 4 and 24 h of incubation, whereas STa had no discernible effect on their expression (Figure 4A). On the other hand, LT regulated the transcript levels of CCL-2 in a different pattern (Figure 4A). At 4 and 24 h, LT treatment suppressed CCL-2 expression, whereas STa did not influence the transcript levels of CCL-2 (Figure 4A). To corroborate these findings, we also assessed cytokine and chemokine release using ELISA. As shown in Figure 4B, LT treatment elicited an increase in the secretion of IL-1β and TNF-α by monocytes at both 4 and 24 h, whereas STa treatment did not affect their secretion. While LT treatment had no effect on CXCL-8 secretion at 4 h, increased CXCL-8 levels were observed in the LT group at 24 h (Figure 4B). Notably, unlike the observed decrease in CCL-2 mRNA expression, LT treatment did not affect CCL-2 secretion by monocytes at either 4 or 24 h. Furthermore, neither LT nor STa significantly affected the secretion of IL-6 after incubation for 4 h or 24 h (Figure 4B).

## Discussion

LT and STa are key virulence factors of ETEC and play crucial roles in ETEC colonization [32, 33] and ETEC-induced diarrhea [26]. However, their effects on the immune system, especially monocytes, are poorly understood. In this study, we found that while STa did not affect monocyte viability or function, LT induced cell death, reduced monocyte phagocytic and intracellular killing activity and inhibited ROS production by monocytes. Moreover, LT stimulated the release of IL-1β, TNF-α, and the chemokines CCL-3 and CXCL-8.

Under healthy conditions, the body maintains a steady number of monocytes through cell proliferation, differentiation, survival, and cell death [16]. This balance can be disturbed by pathogens, some of which have been shown to increase the death of monocytes [27, 28]. In this study, we showed that LT increased monocyte death. These findings are consistent with previous studies showing that LT can induce apoptosis in lymphocytes [34, 35]. Further experiments are necessary to evaluate the cytotoxic effects of STa and LT on other innate immune cells.

The generation of ROS by monocytes is a critical antimicrobial mechanism to control pathogens [36, 37] and serves as a major signalling molecule in various physiological pathways [38, 39]. However, excessive oxidative stress can lead to tissue damage and the development of diseases [40, 41]. While previous studies have shown that LT can induce apoptosis in intestinal epithelial cells by increasing ROS production [42], our findings indicate that neither LT nor STa significantly alters ROS production by monocytes. These results are consistent with those observed in porcine neutrophils, as published in our previous study [21]. Since LT induces cAMP production in both intestinal epithelial cells [43], neutrophils [21] and monocytes, these findings suggest that LT may activate similar signalling pathways in both immune and nonimmune cells but with differing outcomes depending on the cell type. Interestingly, pretreatment with LT inhibited the production of ROS induced by PMA and β-glucans, which are well-known inducers of ROS in porcine monocytes [31, 44]. However, our previous results showed that LT did not affect the ROS production induced by PMA in neutrophils [21]. These findings suggest that LT results in different outcomes in different immune cells, even when similar signalling pathways are activated. Although LT did not induce ROS production in monocytes, our results show that an LT deletion mutant ETEC strain significantly increased ROS production by these cells. Oxidative damage in the intestine of piglets has been reported in previous studies following ETEC infection [45]. Additionally, we found that LT can inhibit ETEC-induced ROS production, suggesting that LT secretion by ETEC may be an effective strategy to counteract ROS-mediated elimination by monocytes. Further experiments could be designed to determine whether the presence of LT might increase ETEC survival in vivo. In fact, many bacterial pathogens can utilize virulence factors, such as catalases of enterohemorrhagic *Escherichia coli* [46] and staphyloxanthin of *Staphylococcus aureus* [47], to resist elimination by immune cells.

Monocytes are professional phagocytes, and phagocytosis is a key innate immune mechanism involved in antibacterial immunity [48]. Our results revealed that LT treatment inhibited the ability of monocytes to phagocytose *E. coli*. These findings concerning the phagocytosis of monocytes are consistent with our previous study showing that LT decreases the uptake of *E. coli* by neutrophils [21]. Virulence factors of other pathogens, such as *Staphylococcus aureus* and enterohemorrhagic *Escherichia coli*, are also known to impair phagocytosis [29, 30]. Our results also revealed that pretreatment with LT inhibited the ability of monocytes to kill intracellular ETECs, which might be connected to the LT-induced inhibition of ROS production by monocytes. Interestingly, our results contrast with those of a previous study in which the murine macrophage line RAW 264.7 was used [14]. This study revealed a 50% decrease in the phagocytosis of ETEC by LT-treated murine macrophages using a gentamicin protection assay. However, on the basis of our results with primary monocytes, which revealed that LT treatment increased the number of live ETECs recovered from these cells, one would expect a similar result with LT-treated murine macrophages. We speculate that this discrepancy might be due to cell- or species-specific differences or technical differences between the studies. Alternatively, minor differences in the amino acid sequence between LTh and porcine (LTp) ETEC strains might lead to subtle differences in their interactions with immune cells. While LTp is conserved between porcine ETEC strains, these strains differ in their capacity to secrete LT [43], and as such, the magnitude of the LT-induced responses might differ.

A key function of monocytes is their capacity to produce cytokines and chemokines in response to different stimuli [49, 50]. These molecules attract and activate various immune cells, playing a central role in immune defense against pathogens. In this study, porcine monocytes exhibited increased secretion of IL-1β, IL-6, and TNF-α after stimulation with LT for 4 and 24 h. Similar trends have also been observed for IL-1β production in mouse dendritic cells [51]. Furthermore, previous studies have shown increased production of IL-1β, IL-6, and TNF-α in human and mouse macrophages in response to stimulation with the B subunit of LT [52]. These findings suggest that further research should investigate whether the B subunit alone is sufficient to induce cytokine production in monocytes. IL-1β, IL-6, and TNF-α are proinflammatory cytokines that can lead to the activation of innate and adaptive immune cells. Furthermore, these proinflammatory factors play crucial roles in the differentiation of monocytes toward macrophages or dendritic cells [53]. Further experiments might be conducted to better characterize the impact of LT on this differentiation process. Our results also revealed that LT treatment significantly increased the expression and production of CCL-3 and CXCL-8. CCL-3 and CXCL-8 are chemokines that attract macrophages and neutrophils, respectively, which can increase inflammation at the infection site to eliminate ETEC. Increased production of CXCL-8 induced by the B subunit of LT has also been observed in human and murine macrophages [52]. Therefore, further research should investigate whether the B subunit is responsible for CXCL-8 production in monocytes stimulated by LT. CCL-2, known as monocyte chemoattractant protein-1, can bind to CCR2 and mediate the recruitment of monocytes [49]. In this study, we observed a discrepancy between CCL-2 transcript and protein levels in monocytes. While LT reduced CCL-2 transcript levels in monocytes at 24 h, it did not affect CCL-2 secretion. One possible explanation is that LT may induce the release of prestored intracellular CCL-2, as previous studies have reported on preformed cytokines in monocytes [54, 55]. Alternatively, LT might activate pathways that increase CCL-2 mRNA translation, counteracting transcriptional suppression, a phenomenon reported previously in humans and pigs [56, 57].

The results revealed that while STa did not affect monocyte viability or function, the enterotoxin LT induced cell death and disrupted the effector functions of porcine monocytes. A main limitation of this study, however, is the presence of low levels of endotoxins in the purified LTs. Despite this, we are convinced that the responses observed in the functional assays are primarily mediated by LT. However, we cannot completely rule out potential additive or synergistic effects of LPS on the LT-induced disruption of monocyte effector functions.

In conclusion, while STa did not affect monocyte viability or function, LT increased the release of inflammatory mediators by monocytes, which may contribute to the clearance of ETEC infections. However, ETEC-derived LT also induced monocyte death and dampened several effector functions of monocytes. Specifically, LT inhibited the ROS production induced by PMA, β-glucan, and ETEC, reduced the uptake of *E. coli*, and enhanced the survival of phagocytosed *E. coli*. These effects likely provide an advantage for ETEC to evade monocyte immune responses and establish infection.

## Data Availability

The data that support the findings of this study are available from the corresponding author upon reasonable request. Source data are provided with this paper.
